# *PIK3CA*-mutations in breast cancer

**DOI:** 10.1007/s10549-022-06637-w

**Published:** 2022-10-24

**Authors:** Kristin Reinhardt, Kathrin Stückrath, Carolin Hartung, Sandy Kaufhold, Christoph Uleer, Volker Hanf, Tillmann Lantzsch, Susanne Peschel, Jutta John, Marleen Pöhler, Marcus Bauer, Friedrich Karl Bürrig, Edith Weigert, Jörg Buchmann, Eva Johanna Kantelhardt, Christoph Thomssen, Martina Vetter

**Affiliations:** 1 Department of Gynaecology, Martin Luther University Halle-Wittenberg, Ernst-Grube-Str. 40, 06120 Halle (Saale), Germany; 2 Gynäkologisch-Onkologische Praxis, Hildesheim, Germany; 3 Department of Gynaecology, Nathanstift, Hospital Fuerth, Fürth, Germany; 4 Department of Gynaecology, Hospital St. Elisabeth and St. Barbara, Halle (Saale), Germany; 5 Department of Gynaecology, St. Bernward Hospital, Hildesheim, Germany; 6 Department of Gynaecology, Helios Hospital Hildesheim, Hildesheim, Germany; 7 Department of Gynaecology, Asklepios Hospital Goslar, Goslar, Germany; 8 Institute of Pathology, Martin Luther University Halle-Wittenberg, Halle (Saale), Germany; 9 Institute of Pathology Hildesheim, Hildesheim, Germany; 10 Institute of Pathology, Hospital Fürth, Fürth, Germany; 11 Institute of Pathology, Hospital Martha-Maria, Halle (Saale), Germany; 12 Institute of Epidemiology, Biometry and Informatics, Martin Luther University Halle-Wittenberg, Halle (Saale), Germany; 13 Present Address: Department of Gynaecology and Obstretrics, Hospital Wolfenbüttel, Wolfenbüttel, Germany; 14 Present Address: Gemeinschaftspraxis Pathologie Amberg, Amberg, Germany

**Keywords:** Early breast cancer, *PIK3CA*, PI3K, Somatic mutations, Prognosis

## Abstract

**Purpose:**

Phosphatidylinositide-3-kinase (PI3K) regulates proliferation and apoptosis; somatic *PIK3CA*-mutations may activate these processes. Aim of this study was to determine the prevalence of *PIK3CA*-mutations in a cohort of early stage breast cancer patients and the association to the course of disease.

**Patients and methods:**

From an unselected cohort of 1270 breast cancer patients (PiA, Prognostic Assessment in routine application, NCT 01592825) 1123 tumours were tested for the three *PIK3CA* hotspot-mutations H1047R, E545K, and E542K by qPCR. Primary objectives were the prevalence of somatic *PIK3CA*-mutations and their association to tumour characteristics. Secondary objective was the association of *PIK3CA*-mutations to recurrence-free interval (RFI) and overall survival.

**Results:**

*PIK3CA*-mutation rate was 26.7% (300 of 1123). *PIK3CA*-mutations were significantly more frequent in steroid hormone-receptor (SHR)-positive HER2-negative (31.4%), and G1 and G2 tumours (32.8%). Overall, we did not observe a significant association of PIK3CA-mutations to RFI. In SHR-positive BCs with PIK3CA-mutations, a strong trend for impaired  RFI was observed (adjusted HR 1.64, 95% CI 0.958–2.807), whilst in SHR-negative BCs *PIK3CA*-mutations were insignificantly associated with improved RFI (adjusted HR 0.49; 95% CI 0.152–1.597). Of note, we observed a significantly detrimental prognostic impact of *PIK3CA*-mutations on RFI in SHR-positive, HER2-negative BCs if only aromatase inhibitors were administered as adjuvant therapy (adjusted HR 4.44, 95% CI 1.385–13.920), whilst no impact was observed in tamoxifen treated patients.

**Conclusion:**

This cohort study speficies the overall mutation rate of PIK3CA in early breast cancer. The impact of PIK3CA-mutations on RFI and OS was heterogeneous. Our results suggest that estrogen deprivation failes to be active in case of PIK3CA-mutation.

**Supplementary Information:**

The online version contains supplementary material available at 10.1007/s10549-022-06637-w.

## Introduction

Phosphatidylinositol-3-kinase (PI3K) (gene symbol *PIK3CA*) intracellularly mediates different processes like promoting cell transformation, tumour initiation and proliferation, and resistance to apoptosis. Its activity is stimulated by extracellular growth factors and hormones [1]. The dysregulation of PI3K initiates activity of the serine/threonine kinase AKT in many cancer entities thereby modulating a range of downstream proteins that promote uncontrolled cellular and tumour growth [2]. Thirty years ago, the PI3K/AKT/mTOR signalling pathway was discovered to be associated with carcinogenesis and oncogenic development [3], as summarized by Arafeh and Samules [4], and to date, *PIK3CA*-targeted drugs are developed and validated in clinical trials [5, 6].

The lipid-based PI3-kinases phosphorylate the 3-hydroxyl group of phosphatidylinositol(4,5)-bisphosphate (PIP2) to phosphatidylinositol(3,4,5)-trisphosphate (PIP3) followed by the activation of AKT and downstream-signaling pathways required for cell growth and survival. PI3K activation is physiologically abrogated by the tumour suppressor phosphatase and tensin-homolog (PTEN) which converts PIP3 back to PIP2. PIP3 peptide levels depend on the competition between PI3K and PTEN. The overactivation of PI3K as well as decreased PTEN expression lead to activated and increased levels of AKT, thus pathologically promoting cell cycle progression [6].

There are three classes of PI3Ks according to their primary structures, substrate preferences and regulation: Class I (Ia, Ib), Class II and Class III. Most relevant for cellular regulation are the PI3Ks of class Ia which act as heterodimers of regulatory and catalytic subunits [7]. The catalytic subunit of the class I PI3-kinase p110α is encoded by the *PIK3CA* gene with a total genomic size of 86,190 base pairs in 21 exons and a final transcript of 3207 base pairs which encode a protein of 1068 amino acids. The p110α protein has five domains: an adaptor-binding-domain for linking the regulatory subunit, a Ras-binding-domain, a C2-domain for binding PIP2 and PIP3, a helical domain and a kinase domain, see https://www.uniprot.org/uniprot/P42336 [8].

Somatic mutations of the *PIK3CA* gene have been described in human cancers in general with a prevelance of up to 40% in primary breast cancer (http://www.sanger.ac.uk/cosmic) [9, 10]. The most frequent *PIK3CA* gene mutations are found in the coding sequence inducing a gain of-function of PI3K. Three hot spot non-synonymous variants represent 87% of the mutations with known clinical relevance [11] leading to amino acid substitutions: COSMIC 760 in exon 9 (17% incidence) with an E545K mutation, COSMIC 763 in exon 19 (17% incidence) affecting E545 and COSMIC 775 in exon 20 (35% incidence) altering H1047.

The three genomic aberrations are predictive for drug responsiveness, meaning that diagnostic testing can identify patients who might benefit from PI3K-targeted therapy. Recently, the PI3K inhibitor alpelisib was approved by both the FDA and EMA for patients with *PIK3CA*-mutated, steroid hormone receptor (SHR)-positive and HER2-negative tumours [5]. The three hotspot mutations described above can be efficiently determined by targeted sequence analysis. Nevertheless, the prognostic and predictive value of the *PIK3CA* mutation status as a biomarker for early breast cancer is discussed controversially for BC subgroups with respect to hormone receptor and HER2 expression [12].

In this study, we describe the prevalence of the three most common *PIK3CA*-mutations in subgroups of a breast cancer cohort and its association with clinical, histopathological characteristics and survival.

## Material and methods

### Patient and tumour characteristics

A prospective study of 1270 early breast cancer patients from five German certified breast centres (2009 to 2011) was designed in accordance with the REMARK (“Reporting Recommendations for Tumor Marker Prognostic Studies”) criteria [13] and registered as the “PiA-study” [14] (Prognostic assessment in routine application, NCT 01592825) using the following inclusion criteria: female patients, aged 18 years or older, invasive, non-metastatic BC and no secondary cancer, no limitation in tumour size, lymph node involvement, and grading or expression of estrogen receptor (ER), progesterone receptor (PgR) and human epidermal growth factor receptor 2 (HER2). Patients were diagnosed and treated (1070 with primary surgery, 200 with neoadjuvant chemotherapy, NACT) according to the annually updated German AGO Guidelines (AGO) valid at the respective times https://www.ago-online.de/leitlinien-empfehlungen/leitlinien-empfehlungen/kommission-mamma.^41^

**Fig. 1:**
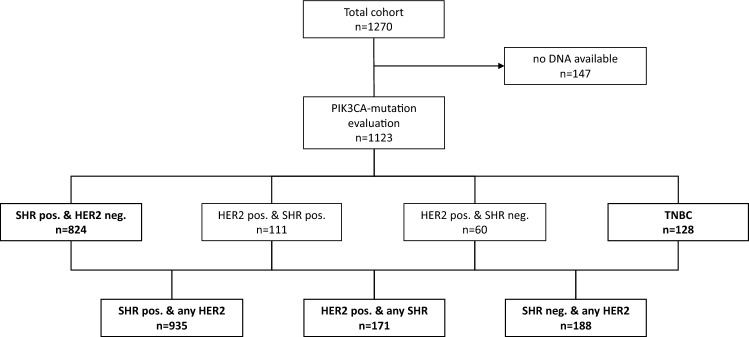
Enrolment of patients of the PiA-cohort (*n* = 1270) and groups that were used for multivariate *PIK3CA*-mutation analyses (*n* = 1123) (bold)

In the current study, we analysed the *PIK3CA* gene mutation status of 1123 tumours. Median age of the patients was 60 years at time of diagnosis, with three-quarters of patients being older than 50 years, and two thirds having no lymph node involvement. Considering tumour tissue, three-quarters were well differentiated or intermediate (G1 or G2), and half of the tumours were smaller than 2 cm. The distribution of the patients' main characteristics, as well as the histopathological parameters of the analysed cohort (*n* = 1123), did not significantly differ from the entire PiA cohort (supplementary table S 1). The enrolment of the patients and grouping for the main analysis is shown in Fig. 1.

### Endpoints and statistical analysis

We defined the prevalence of *PIK3CA*-mutations and the associations between *PIK3CA* mutation status and clinical histopathological parameters as first objective, applying binary logistic regression; recurrence free interval (RFI) and overall survival (OS) were the second objectives. RFI-related events refer to local invasive recurrence, distant recurrence, and death from breast cancer. OS included death from breast cancer, non-breast cancer causes, and unknown causes [15]. Tumour association and survival analysis were only performed for patients with one tumour. Considering the risk of multiple testing, we reduced the subgroup analyses to pre-defined, well-accepted and clinical relevant groups (e.g., tumour size, nodal status, grading, IHC-types, type of treatment etc.). The median observation time after diagnosis was 62 months (1–132). The impact of *PIK3CA* mutation status was calculated using Kaplan–Meier estimates; differences were described by log-rank test and multivariate analyses for selected parameters were carried out applying proportional hazard regression model and a fixed effects model (Review Manager, version 5.3). Recursive partitioning by **C**lassification **a**nd **R**egression **T**ree (CART) analysis was performed to examine combinations of clinical and histopathological parameters to find homogenous risk groups with respect to RFI [16]. To minimize selection bias for any parameter, only significant variables were entered into the CART analysis starting with the most important prognostic parameter resulting from regression model. Patient groups were further recursively divided, considering the best split at each decision point into smaller and more homogenous groups. Unbiased parameter selection was guaranteed by following the best impact parameter (after regression model) [17].

All statistical tests were two-sided, and a p-value below 0.05 was considered to be significant. Statistical analyses were carried out using SPSS 25 (IMB, Armonk, NY, USA).

### DNA extraction and hotspot mutation assay

Fresh frozen tissue (FF) (*n* = 813) and formalin-fixed, paraffin-embedded tumour material (FFPE) (*n* = 310) of the tumours were used for DNA isolation [18]. The FF-tissue was dismembrated in liquid nitrogen, and powder was used for DNA extraction using the QIAamp DNA Mini Kit [19] (Cat. No. 51304; Qiagen, Hilden, Germany). For isolation of DNA from FFPE specimens, 3–5 adjacent unstained tumour slices (5 µm) were processed using QIAamp DNA FFPE Tissue kit (Cat. No. 56404; Qiagen, Hilden, Germany). All preparations were performed according to the manufacturer’s instructions. Quality and concentration of the extracted DNA were assessed with a Tecan Infinite PRO® 200 (Tecan, Männedorf, Switzerland). A standard amount of 50 ng DNA was subjected to mutation analysis.

With respect to mutation status, we focused on the three most common hotspot mutations COSMIC C775 (H1047R), C763 (E545K) and C760 (E542K). TaqMan® Mutation Detection and reference assays were performed in duplicates (Life Technologies, Carlsbad, CA, USA) and were used for quantitative PCR (qPCR) with the StepOne Plus® Real-Time PCR System (Life Technologies) for 40 cycles with 60 °C amplification temperature.

## Results

### PIK3CA mutation prevalence and association with clinical and histopathological parameters

In this cohort study, 88% of the patients were tested for single nucleotide substitutions at three hotspot positions in the *PIK3CA* gene, and we found a mutation prevalence of 26.7% (*n* = 300 of 1123) considering these positions. The mutation rates for the three hot spot sites were 58% (*n* = 174) for COSMIC C775 (H1047R), 28% (*n* = 85) for C763 (E545K) and 14% (*n* = 43) for C760 (E542K). Co-occurrence of mutations at C775 and C763 were found in two tumours. *PIK3CA*-mutations were significantly more frequent in well and intermediately compared to poorly differentiated tumours (G1, OR 3.13, 95% CI 1.970–4.986; G2, OR 2.14, 95% CI 1.478–3.085). PIK3CA-mutations were significantly more often observed in steroid hormone receptor-positive than in steroid hormone receptor-negative tumours (OR 3.38, 95% CI 2.103-5.438), and in HER2-negative than in HER2-positive tumours (OR=2.25, 95% CI 1.451-3.501), respectively. We combined SHR- and HER2-status and found that the HER2-negative luminal-like IHC-type (*n* = 259 of 824, 31.4%) had the highest occurrence of *PIK3CA*-mutations (OR 4.13, 95% CI 1.753–9.712). Only 11.7% of TNBC tumours harboured a *PIK3CA*-mutation (15 of 128). There was no significant association with age and nodal status. The prevalences of *PIK3CA*-mutations in selected subgroups are reported in Table 1.

**Table 1:** *PIK3CA*-mutation prevalence (%) in selected clinical and histopathological groups

Parameters	PIK3CA-cohort	PIK3CA n	mutated (prevalence)	Odds Ratio	95% CI	p-value
All	1123	300	(26.7%)			
Age at time of diagnosis						
≤ 50 years	293	68	(23.2%)	1		
> 50 years	830	232	(28.0%)	1.284	0.941–1.751	0.115
Histological type						
Ductal	905	241	(26.5%)	2.132	0.993–4.578	0.058
Lobular	163	51	(31.3%)	2.675	1.179–6.071	0.019
Others	55	8	(14.5%)	1		
Tumour size at time of diagnosis						
< 2 cm	575	172	(23.0%)	1.4	1.073–1.828	0.013
≥ 2 cm	548	128	(18.9%)	1		
Nodal status at time of diagnosis						
negative	688	180	(26.2%)	1		
positive	435	120	(27.6%)	1.075	0.820–1.407	0.599
Tumour differentiation						
G1	154	57	(37.0%)	**3.134**	1.970–4.986	< 0.000
G2	703	201	(28.6%)	**2.135**	1.478–3.085	< 0.000
G3	266	42	(15.8%)	1		
Estrogen receptor status						
positive (≥ 1%)	919	278	(30.3%)	**3.588**	2.255–5.708	< 0.00
negative (< 1%)	204	22	(10.8%)	1		
Progesterone receptor status						
positive (≥ 1%)	776	245	(31.6%)	**2.45**	1.769–3.392	< 0.000
negative (< 1%)	347	55	(15.9%)	1		
Steroid hormone receptor status						
positive	935	279	(29.8%)	**3.382**	2.103–5.438	< 0.000
negative	188	21	(11.2%)	1		
HER2 status						
negative	952	274	(28.8%)	**2.254**	1.451–3.501	< 0.000
positive	171	26	(15.2%)	1		
IHC-types						
SHR-positive and HER2-negative	824	259	(31.4%)	**4.126**	1.753–9.712	0.001
HER2-positive and SHR-positive	111	20	(18.0%)	1.978	0.748–5.231	0.169
HER2-positive and SHR-negative	60	6	(10.0%)	1		
TNBC	128	15	(11.7%)	1.195	0.439–3.250	0.728

*CI* confidence interval, SHR steroid hormone receptor, HER2 human epidermal growth factor receptor 2, TNBC triple-negative breast cancer

Bold: significant in prognostic parameters

###  Association between *PIK3CA* mutation status and survival

**Fig. 2:**
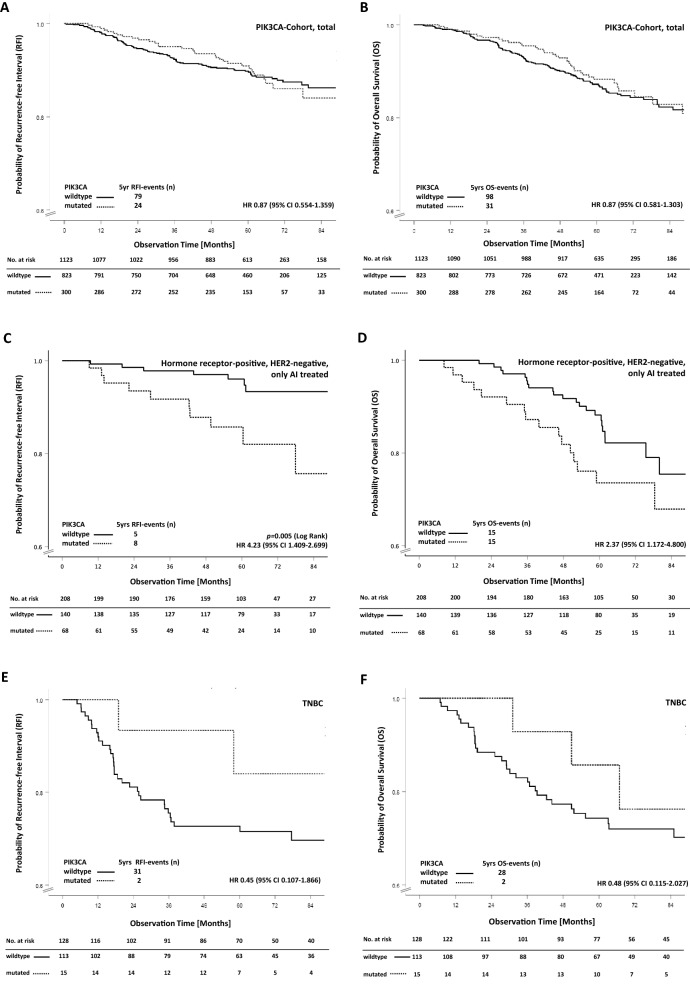
Survival estimates for RFI and OS stratified by detection of PIK3CA-mutations. The tables present the effective sample size for each interval (numbers at risk). **A**, **B** All patients (*n* = 1123), RFI (**A**) and OS (**B**). **C**, **D** Patients with SHR-positive and HER2-negative tumours, Aromatase Inhibitors (AI) treatment (*n* = 208), RFI (**C**) and OS (**D**). **E**, **F** Patients with SHR-negative and HER2-negative tumours (TNBC) (*n* = 128), RFI (**E**) and OS (**F**)

Overall, we did not observe any significant association between presence of *PIK3CA*-mutations and RFI (event-free at 5 years 90.9% for mutated, 89.9% for wildtype; adjusted HR 1.19, 95% CI 0.752–1.894, Fig. 2A) and OS (alive at 5 years 88.2% for mutated, 87.2% for wildtype; adjusted HR 1.08, 95% CI 0.714–1.638, Fig. 2B), neither in univariate nor multivariate analyses (Table 2).

**Table 2 Comment: the header Overall Survival is missing, please exchange with the attached file:** Univaraite and multivariate analyses of RFI and OS for all patients with regard to selected parameters

Parameter	Sample size *n* = 1123	Recurrence free interval, RFI (103 events)											
		Univariate analysis			Multivariate analysis			Univariate analysis			Multivariate analysis		
		HR	95% CI	p- value	HR	95% CI	p- value	HR	95% CI	p- value	HR	95% CI	p- value
Age													
≤ 50 years	293	1.492	0.996–2.235	0.052				1					
> 50 years	830	1						1.536	0.993–2.375	0.054			
Tumour size at time of diagnosis													
< 2 cm	575	1			1			1			1		
≥ 2 cm	548	3.834	2.429–6.054	< 0.000	2.417	1.498–3.898	< 0.000	3.530	2.358–5.284	< 0.000	2.506	1.642–3.826	< 0.000
Nodal status at time of diagnosis													
negative	688	1			1			1			1		
positive	435	3.074	2.059–4.589	< 0.000	2.045	1.347–3.104	0.001	2.643	1.856–3.764	< 0.000	1.882	1.303–2.718	0.001
Tumour differentiation													
G1	154	1			1			1			1		
G2	703	14.395	1.995–103.8	0.008	4.485	1.085–18.536	0.038	2.406	1.107–5.231	0.027	1.570	0.713–3.459	0.263
G3	266	27.149	3.736–197.2	0.001	5.241	1.224–22.445	0.026	4.772	2.168–10.506	< 0.000	2.310	1.005–5.308	0.049
Steroid hormone receptor status													
negative	188	4.449	3.019–6.556	< 0.000	3.178	2.056–4.915	< 0.000	2.676	1.857–3.857	< 0.000	1.768	1.177–2.657	0.006
positive	935	1			1			1			1		
HER2 status													
negative	952	1			1.080	0.668–1.748	0.752	1			1		
positive	171	1.482	0.926–2.372	0.101	1			1.606	1.063–2.428	0.024	1.160	0.760–1.772	0.492
*PIK3CA* mutation status													
wildtype	823	1			1			1			1		
mutuated	300	0.867	0.554–1.359	0.535	1.193	0.752–1.894	0.454	0.870	0.581–1.303	0.5	1.081	0.714–1.638	0.712

Within the cohort of patients with positive steroid hormone receptor status (irrespective of HER2 status, *n* = 935), 8.5% of the patients with *PIK3CA*-mutations experienced RFI events within 5 years of follow-up compared to 6.2% with *PIK3CA*-wildtype (adjusted HR 1.64, 95% CI 0.958–2.807, *p* = 0.071). Overall survival probability at 5 years was 88.1% and 90.5%, respectively (adjusted HR 1.37, 95% CI 0.867–2.152). We found numerically more RFI events at 5 years in patients with SHR-positive, HER2-negative and *PIK3CA*-mutated tumours than in patients with *PIK3CA*-wildtype tumours (7.9% and 6.0%, resp., Fig. S1A). More patients in this group died if their tumours were *PIK3CA*-mutated (11.4% and 8.5%, resp., Fig. S1B). However, the effect was not significant, neither in univariate, nor in multivariate analyses.

Of note, patients with SHR-positive HER2-negative tumours who were treated with aromatase inhibitors only (*n* = 208), had a significant 4.39 times higher occurrence of RFI events if they harboured a *PIK3CA*-mutation (*n* = 68) compared to those with *PIK3CA*-wildtype (*n* = 140; adjusted HR 4.39, 95% CI 1.385–13.920, *p* = 0.012; Fig. 2C, Table S3A) and a significantly impaired OS (adjusted HR 2.12, 95% CI 1.021–4.404, *p* = 0.044; Fig. 2D, Table S3B). In contrast, no association between *PIK3CA* mutation status and RFI or OS was observed in patients with luminal-like tumours who were treated with tamoxifen only (Fig. S1C/D, Table S3A/B).

In the hormone receptor-negative group (irrespective of HER2 status), patients with *PIK3CA*-mutated tumours (*n* = 21 of 188, 11.2%) showed numerically fewer RFI-events (3 of 21 vs 43 of 167) consistent with a higher RFI probability (84.4% and 72.9%, resp.; adjusted HR 0.49, 95% CI 0.152–1.597; Fig. S1E). In contrast, in TNBC, numerically, more patients with PIK3CA-mutations (84%) were free of RFI-events after 5 years than those with wildtype *PIK3CA* (71.5%; adjusted HR 0.43, 95% CI 0.103–1.822, Fig. 2E). These observations were similar after exclusion of patients without adequate (neo)adjuvant therapy. For the HER2-positive group (any SHR), we did not observe any significant impact of *PIK3CA*-mutations on RFI or OS (Fig. S1G/H). Interestingly, patients with SHR-negative tumours experienced a better overall survival if a *PIK3CA-*mutation was detected (Fig. 2F, Fig. S1F,H). The different impact of *PIK3CA-* mutation status on RFI in relation to steroid hormone receptor- and HER2-expression is visualized in the corresponding forest plot (Fig. S2).

**Fig. 3:**
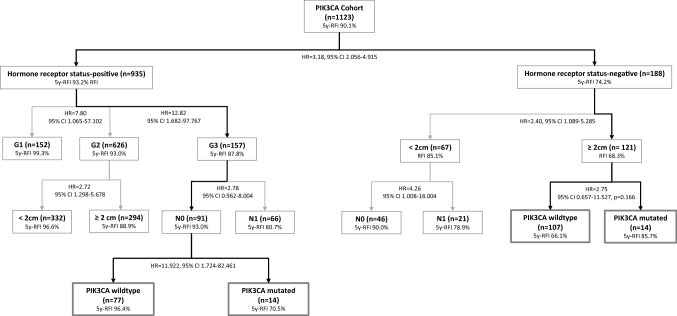
Classification and Regression Tree (CART) for PIK3CA mutations. Bold arrows indicate the clinical value of PIK3CA mutations in subgroups

To identify homogenous risk groups with regard to *PIK3CA* mutation status, we used a recursive partitioning procedure (Fig. 3). In node-negative, SHR-positive undifferentiated (G3) tumours, patients with a *PIK3CA*-mutation (*n* = 14) had a worse 5 year-RFI (70.5%) than those with wildtype *PIK3CA* (5 year RFI 96.4%, HR 11.92; 95% CI 1.724–82.461, *p* = 0.012). In contrast, in SHR-negative larger tumours (≥ 2 cm), patients with *PIK3CA-* mutations (*n* = 14) showed a trend to better 5 years-RFI probability (85.7%) compared to those with wildtype PIK3CA (5 years RFI 66.1%, HR 2.75; 95% CI 0.657–11.527). However, the absolute survival differences are substantial for each group and might in total be relevant for 18.9% of all patients (SHR-pos. G3 pN0, *n* = 91, and SHR-neg. pT2, *n* = 121).

### Association of PIK3CA mutation status to pathologic complete response (pCR) rates

The *PIK3CA* mutation status was available for 120 tumour samples of TNBC patients receiving NACT, 20 of them with a *PIK3CA-*mutation. Due to the small numbers, only a trend was observed indicating that patients with wildtype tumours achieved pCR more often than patients with PIK3CA-mutations. No subgroup analysis was possible.

## Discussion

In this study, to our knowledge we present the largest analysis of *PIK3CA* oncogenic mutations to date, using an unselected routine cohort of early stage breast cancer patients (*n* = 1123). The principal aim of our study was to evaluate the prevalence of *PIK3CA-*mutations and their associations with clinical and histopathological parameters and secondary the relation of a *PIK3CA-*mutation to clinical outcome. Since the landmark study of Samuels et al. [20], it has been known that presence of somatic *PIK3CA*-mutations promotes cancer progression also in breast cancer. Most previous publications have reported on heterogeneous sample sets including mixed sets of early stage and metastatic breast cancer patients from retrospective studies. Instead, our findings of a prospective well-defined homogenous cohort of early-stage breast cancer patients provide new insights to the realistic frequency of *PIK3CA-*mutations overall and in subgroups, as well as their association with recurrence-free interval and overall survival.

We detected an overall somatic mutation rate of 26.7% (300 of 1123 samples) when testing the three most common hot spots C775 (H1047R), C763 (E545K), and C760 (E542K) (https://www.mycancergenome.org/), which represent 87% of all mutations currently known in the *PIK3CA* gene [11]. Of interest, the highest frequencies (> 30%) of *PIK3CA* gene mutations were detected in tumours with more favourable characteristics (G1, G2, ER-positive, PgR-positive, luminal-like, HER2-negative), which is in line with most other studies and available data in the COSMIC database http://www.sanger.ac.uk/cosmic/http://www.sanger.ac.uk/cosmic/ [21]. In tumours with high risk biology (HER2-positive, TNBC) we found the lowest rate of *PIK3CA*-mutations (15.2% and 11.4), which is also consistent with published data [11].

These findings provoke the question why *PIK3CA-*mutations are more frequently detected in ER-positive disease. The current state of research postulates that *PIK3CA*-mutation-dependent activation of AKT phosphorylates and activates ER leading to transcriptional activity of ER in an oestradiol-independent manner and consecutively to preferential growth of ER-positive cancer [22, 23]. Thus, mutated PI3K likely promote ER-positive cancer growth and may explain the overrepresentation of *PIK3CA*-mutated tumours in luminal and well-differentiated breast cancer. In addition, *PIK3CA*-mutations are considered an early event in breast cancer development since they were detected even in small tumours as well as in non-invasive precursor lesions, like DCIS [24]. In contrast, fast growing ER-negative and undifferentiated tumours, however, may be derived from different precursor cells and independent of activating *PIK3CA*-mutations.

### Prognostic and predictive implications

The second objective of our study was the prognostic impact of *PIK3CA-*mutations, and we did not find any association with recurrence free interval (RFI) or overall survival (OS) within the entire cohort of 1123 patients. We choose RFI as endpoint since we wanted to analyse the clean disease-related impact of *PIK3CA*-mutations. We tested an unselected and rather large cohort of early breast cancer patients, thus we assume that our data provide a realistic view, demonstrating a lack of a general impact of *PIK3CA*-mutations on the course of disease in breast cancer. This is in contrast to the published meta-analyses and single studies on *PIK3CA-*mutations that present conflicting results on its association to prognosis; studies found an association to better survival (e.g. Dumont et al., Pang et al. [25, 26]) as well as to inferior survival (Sobhani et al., Fan et al.) [12, 27]. These divergent results might presumably result from the heterogeneity of the populations that were studied with regard to sample size, subgroups, and type of treatment, so, selection bias cannot be excluded in these analyses (for review see [28]).

The published results are also inconclusive with regard to a potential predictive impact of *PIK3CA*-mutations. However, in our study we found a significant predictive value of *PIK3CA*-mutations in luminal breast cancer by observing more disease-related events in patients with *PIK3CA*-mutations. Most importantly, there was an association to the type of endocrine therapy: We found a significant impact of PIK3CA-mutations on the effect of adjuvant aromatase inhibitors, but no impact on the effect of adjuvant tamoxifen. This observation may be explained by PI3K-triggered estradiol-independent activation of the ER that can be observed in estradiol-deprived situations created by aromatase inhibition but might be blocked by ER-modulation through tamoxifen as postulated by Campbell and colleagues [22]. This differential therapy response has also been described for advanced BC by Ramirez-Ardila et al. [29].

It has to be acknowledged that these relations are complex and other mechanisms are involved. For example, recent findings suggest that PI3K pathway alterations might be associated with the composition of the tumour microenvironment in luminal breast cancer, including the attraction of CD8-positive T-cells [30]. Our observations are fully in line with the data of Stemke-Hale and colleagues who also did not find an association between *PIK3CA*-mutations and the effect of adjuvant tamoxifen [31]. However, data are again heterogeneous; some authors described resistance to tamoxifen [32, 33], whilst others found significantly improved endocrine sensitivity to tamoxifen if *PIK3CA*-mutations were detected [34].

The results from our observational cohort study support the finding that *PIK3CA*-mutations may indicate resistance to aromatase inhibitor therapy; however, prospective studies are lacking.

In patients with HER2-positive breast cancer we found no impact of *PIK3CA*-mutations on RFI. Similarly, a well described pooled analysis of five prospective clinical trials showed no significant impact of *PIK3CA*-mutation on the course of disease in patients with adequately treated HER2-positive BC, although the *PIK3CA*-mutated group had a significantly lower pCR rate [35]. In an uniformly treated early-stage HER2-positive Danish cohort, the *PIK3CA-*alterations predicted a significantly worse OS (adjusted HR 2.14), but had no significant impact on invasive disease free survival (iDFS) presumably due to the small sample size [36].

An exploratory analysis of the CLEOPATRA trial identified a subgroup of HER2-positive *PIK3CA*-mutated patients who were resistant to anti-HER2-therapy with trastuzumab and pertuzumab (worse OS if mutated, adjusted HR 1.48, *p* = 0.0025) [37]. Contradictory results may be explained by the continued activation of PI3K and an inhibitory effect on HER2 signaling [38]. Thus, in patients with HER2-positive tumours the impact of *PIK3CA*-mutations is not clear; at least the effects are small and not significant.

The presence of *PIK3CA*-mutations may have a favourable impact in early TNBC, suggested by a 2.3-times improved RFI and a 3-times improved OS, which is in line with Mosele and Takeshita, even though they worked with samples from advanced BC [39] analysing cell-free DNA [40].

To the best of our knowledge, this is the first study using CART in order to find out if combinations of variables could predict the risk of an RFI event. For nearly one fifth of the patients the gene modifications seem to have a relevant prognostic impact depending on the SHR status of the tumour. Overall, using the CART algorithms (see Fig. 3) for 121 of 1123 patients, the presence of gene alterations predict a worse prognosis in defined subgroups. Patients with *PIK3CA*-mutated tumours (*n* = 20) had lower pCR rates than wildtype tumours. This observation is similar to other studies [35].

Our real world data from a multicentre cohort adds exploratory, but valuable information, as our patients were consecutively enrolled in the daily clinical routine.

## Conclusion

The real overall somatic mutation rate of *PIK3CA* is 26.7% when testing the three most common hot spots H1047R, E545K, and E542K in a representative cohort of patients with early breast cancer. We did not find an impact of *PIK3CA*-mutation on RFI and OS in general. As clinical relevant result, we demonstrated resistance of early breast cancer with somatic *PIK3CA*-mutation to adjuvant aromatase inhibitor therapy, suggesting tamoxifen as preferred therapy in these patients. Though only exploratory, this observation is in line with previous observations in metastatic disease. More functional studies are needed to understand the interactions and crosstalk between the activated PI3K signaling pathway and tumorigenesis.

### Strength and limitations

The first intent of our study was to describe the prevalence of presumably prognostic and predictive factors including *PIK3CA*-mutation in the daily routine. The analysis of a prospectively collected and unselected cohort of non-metastatic breast cancer patients with a huge sample size is the strength of our *PIK3CA* study. Limitations may be relevant for the second endpoint of this study exploiting the prognostic and predictive impact of *PIK3CA*-mutations since systemic treatment was slightly heterogeneic although patients were treated in high level certified breast centres and treatment decisions were made according to national guidelines. However, it represents rather a real world situation and cannot be compared to the homogeneity of treatment that is defined by prespecified clinical trial inclusion criteria. In addition, it has to be mentioned, that during the time of enrolment the detection limit for endocrine sensitive tumours was modified on a national and international level; therefore, today we would have a higher proportion of endocrine-treated patients.

We are well aware that subgroup analyses always include the risk of type 1 error for multiple testing. We, therefore, reduced the analyses to prespecified clinical relevant and broadly accepted subgroups and used multivariate analyses to limit the risk of multiple testing.

Another limitation is that as in most published studies, we analysed only the three most frequent *PIK3CA* “hot spot” mutations, such that a small underrepresentation of the total number of mutations may be possible.

## Supplementary Information

Below is the link to the electronic supplementary material.

Supplementary file1 (PDF 1270 kb)

## Data Availability

The data generated in this study are available within the article and its supplementary data files. Raw data were generated and processed from the authors and are available on request to the corresponding author.
