# Supplementary material for: PIK3CA-mutations in breast cancer
Source: Breast Cancer Res Treat. 2022 Oct 24;196(3):483–93. doi: 10.1007/s10549-022-06637-w (PMC9633529; doi:10.1007/s10549-022-06637-w)
Supplement: Supplementary file 1 — Supplementary file1 (PDF 1270 kb) [file 10549_2022_6637_MOESM1_ESM.pdf]

PIK3CA mutations in breast cancer

Kristin Reinhardt[a], Kathrin Stückrath[a], Sandy Kaufhold[a], Christoph Uleer[b], Volker Hanf[c], Tillmann Lantzsch[d], Susanne Peschel[e], Jutta John[f], Marleen Pöhler[g], Marcus Bauer[h], Friedrich Karl Bürrig[i], Edith Weigert[j], Jörg Buchmann[k], Eva Johanna Kantelhardt[a,m], Christoph Thomssen[a], Martina Vetter[a]*

Corresponding author

Dr. rer. nat. Martina Vetter

Department of Gynaecology, Martin Luther University Halle Wittenberg

Ernst-Grube-Str. 40

06120 Halle (Saale), Germany

Tel. +49 345 5571336

E-mail: martina.vetter@uk-halle.de

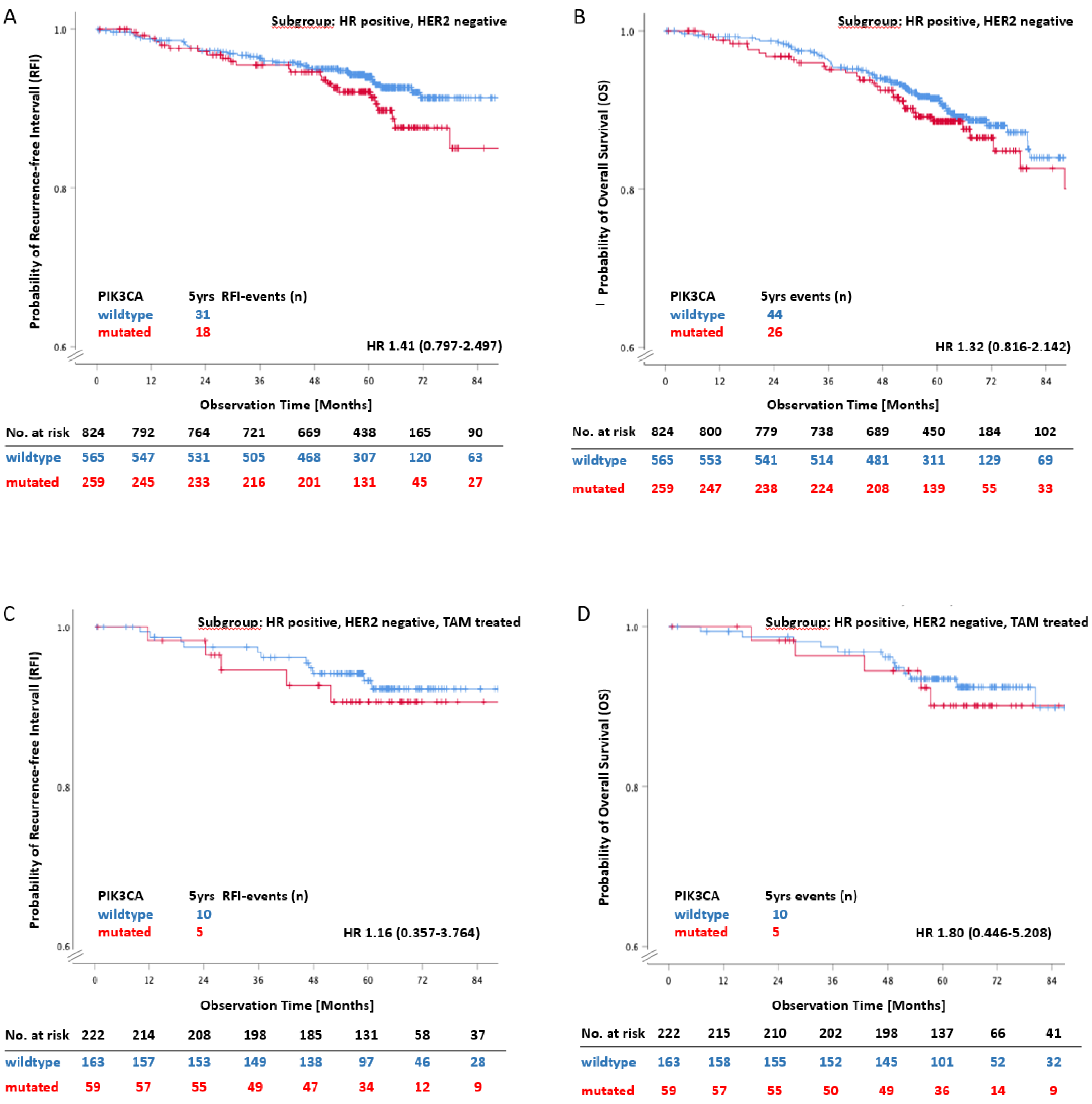

Supplement Fig. S1 Survival estimates for RFI and OS stratified by detection of PIK3CA- mutations.
The tables present the effective sample size for each interval (numbers at risk).

A, B: Patients with HR positive and HER2 negative tumours (n=824), RFI (A) and OS (B)

C, D: Patients with HR positive tumours and HER2 negative tumours, Tamofixen treatment only (n=222), RFI (C) and OS (D)

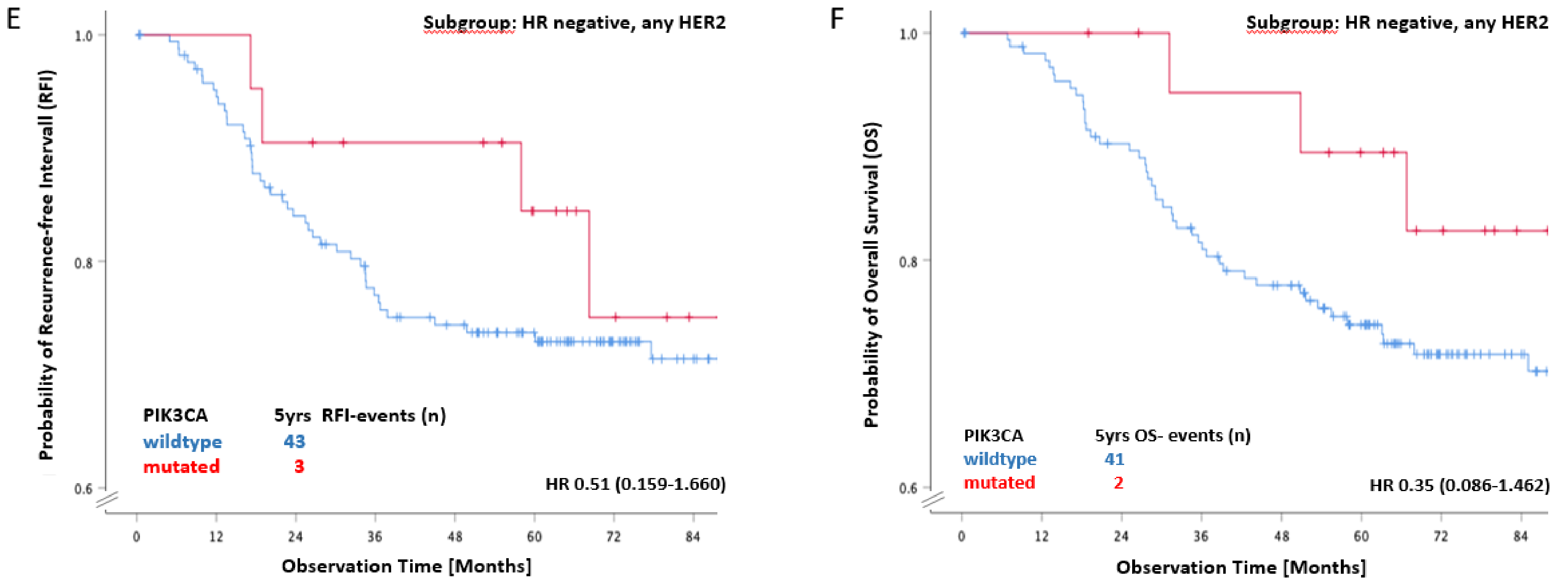

No. at risk	188	188	174	153	135	128	103	65
wildtype	167	167	154	134	118	111	91	58
mutated	21	21	20	19	17	17	12	7

No. at risk	186	180	166	147	138	112	73	56
wildtype	166	160	146	129	120	97	63	49
mutated	20	20	20	18	18	15	10	7

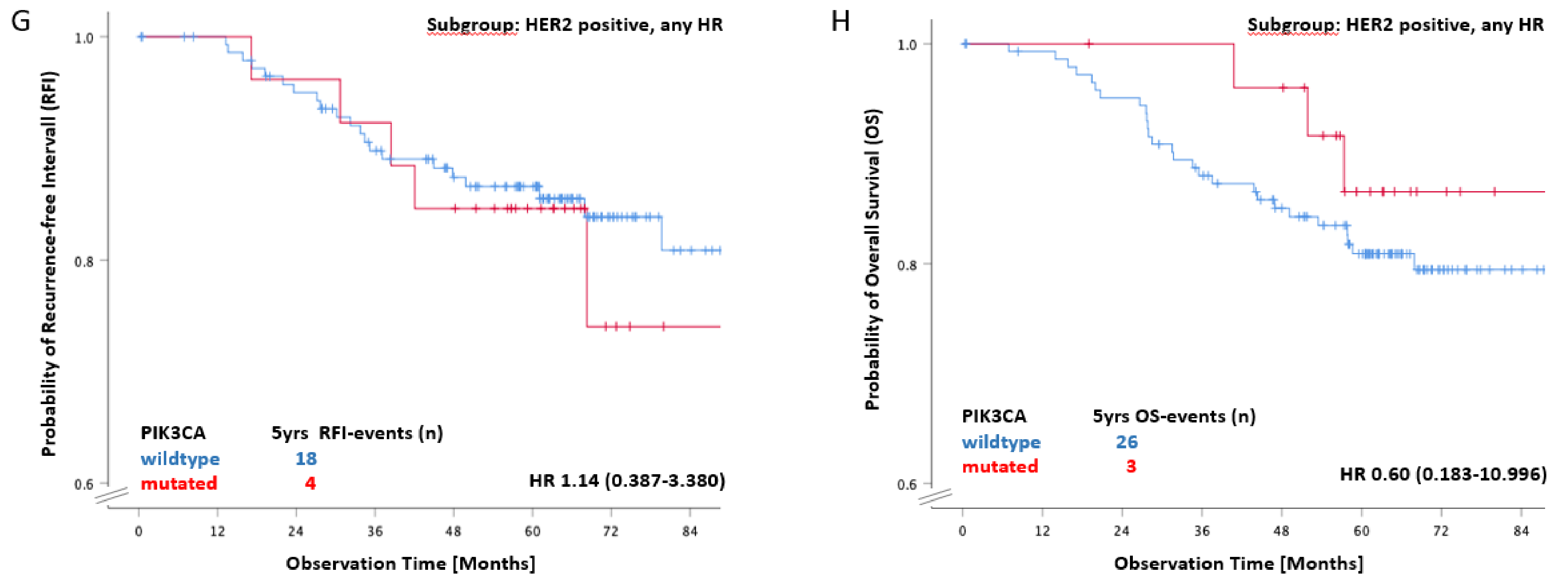

No. at risk	171	166	156	142	126	103	44	27
wildtype	145	141	131	118	105	88	39	24
mutated	26	25	25	24	21	15	5	3

No. at risk	171	166	160	147	133	106	51	36
wildtype	145	141	135	122	110	91	43	30
mutated	26	25	25	25	23	15	8	6

Supplementary Fig. S1 Survival estimates for RFI and OS stratified by detection of PIK3CA- mutations
The tables present the effective sample size for each interval (numbers at risk)

E, F: Patients with HR negative tumours, irrespective of HER2 status (n= 188), RFI (C) and OS (D)

G, H: Patients with HER2 positive tumours, irrespective of HR status (n=171), RFI (G) and OS (H)

Study or Subgroup	log[Hazard Ratio]	SE	Number Total	Events Total	Weight	Hazard Ratio IV, Fixed, 95% CI	Year
**2.1.1 Hormone Receptor Status**							
HR positive	0.495	0.274	935	58	82.7%	1.64 [0.96, 2.81]	2001
HR negative	-0.707	0.6	188	46	17.3%	0.49 [0.15, 1.60]	2002
**Subtotal (95% CI)**			**1123**	**104**	**100.0%**	**1.33 [0.82, 2.17]**	
Heterogeneity: Chi² = 3.32, df = 1 (P = 0.07); I² = 70%							
Test for overall effect: Z = 1.15 (P = 0.25)							
**2.1.2 Receptor Groups**							
HR positive HER2 negative	0.427	0.294	824	49	69.7%	1.53 [0.86, 2.73]	2001
HER2 positive any HR	0.418	0.562	171	22	19.1%	1.52 [0.50, 4.57]	2002
TNBC	-0.834	0.732	128	33	11.2%	0.43 [0.10, 1.82]	2003
**Subtotal (95% CI)**			**1123**	**104**	**100.0%**	**1.33 [0.82, 2.15]**	
Heterogeneity: Chi² = 2.63, df = 2 (P = 0.27); I² = 24%							
Test for overall effect: Z = 1.16 (P = 0.25)							

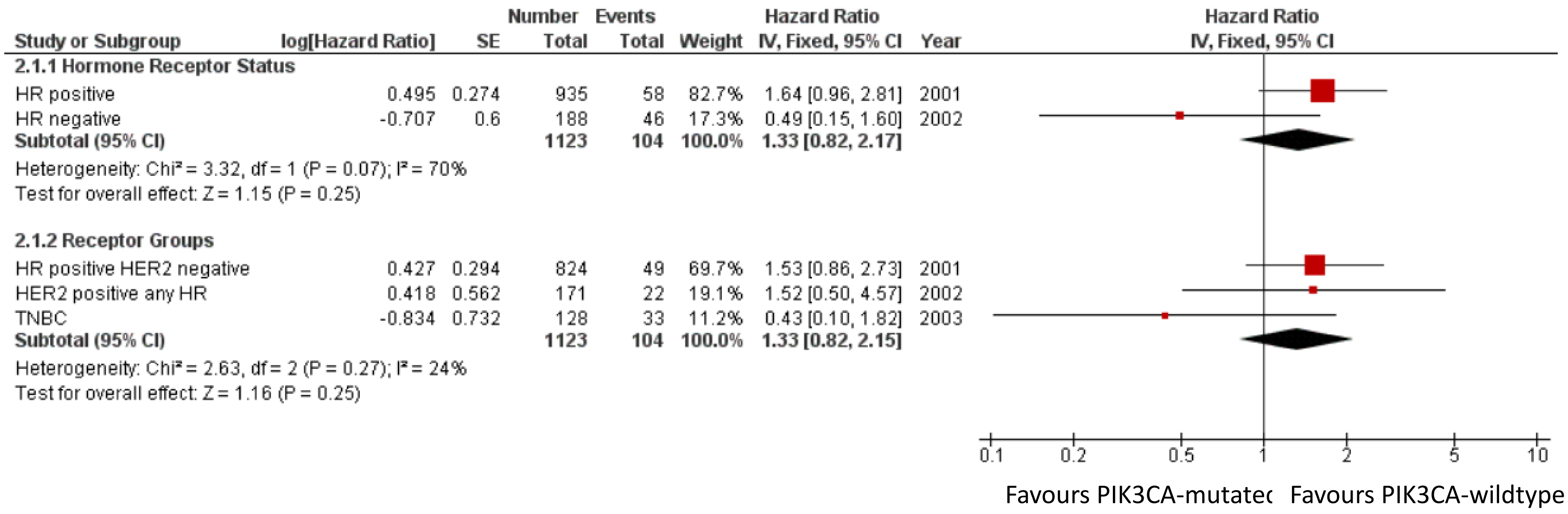

Supplementary Fig. S2 Forest plot for the effect of PIK3CA mutation in different subgroups on RFI
Hazard ratios for subtotals represent aggregation of the weighted subgroup, therefore results that may be different from calculations based on indvidual data

Supplementary Table S1 Patients characteristics and histopathological parameters of the tumours with PIK3CA mutations

Parameters	Total cohort n=1,270 (100 %)	PIK3CA - cohort n=1,123 (100%)
**Age at time of diagnosis**		
≤ 50 years	344 (27.1%)	293 (26.1%)
> 50 years	926 (72.29%)	830 (73.9%)
**Histological type**		
ductal	1039 (81.8%)	905 (80.6%)
lobular	172 (13.5%)	163 (14.5%)
others	59 (4.7%)	55 (4.9%)
**Tumour size at time of diagnosis**		
< 2cm	636 (50.1%)	575 (51.2%)
≥ 2 cm	634 (49.9%)	548 (48.8%)
**Nodal status at time of diagnosis**		
Node-negative	780 (61.4%)	688 (61.3%)
Node-positive	490 (38.6%)	435 (38.7%)
**Tumour differentiation**		
G1	167 (13.1%)	154 (13.7%)
G2	794 (62.5%)	703 (62.6%)
G3	309 (24.4%)	266 (23.6%)
**ER status**		
ER positive (≥ 1%)	1,020 (80.3%)	919 (81.8%)
ER negative (< 1%)	250 (19.7%)	204 (18.2%)
**PgR status**		
PgR positive (≥ 1%)	862 (67.9%)	776 (69.1%)
PgR negative (< 1%)	408 (32.1%)	347 (30.9%)
**HR status (ER, PgR combined**)		
HR positive	1,038 (81.7%)	935 (83.3%)
HR negative	232 (18.3%)	188 (16.7%)
**HER2 Status**		
HER2 negative	1,060 (83.5%)	952 (84.8%)
HER2 positive	210 (16.5%)	171 (15.2%)
**IHC types**		
HR positive and HER2 negative	907 (71.4%)	824 (73.4%)
HER2 positive and HR positive	133 (10.5%)	111 (9.9%)
HER2 positive and HR negative	78 (6.1%)	60 (5.3%)
TNBC	152 (12.0%)	128 (11.4%)

Supplementary Table S2 Patients characteristics and histopathological parameters of the tumours for patients treated with Aromatase Inhibitors (n=208)

Parameters	AI treated - cohort n=208 (100%)	Wildtype - cohort n=140 (100%)	PIK3CA - cohort n=68 (100%)
**Age at time of diagnosis**			
≤ 50 years	1 (0.5%)	1 (0.7%)	- -
> 50 years	207 (99.5%)	139 (99.3%)	68 (100.0%)
**Histological type**			
ductal	159 (76.4%)	101 (72.1%)	58 (85.3%)
lobular	30 (14.4%)	22 (15.7%)	8 (11.8%)
others	19 (9.1%)	17 (12.1%)	2 (2.9%)
**Tumour size at time of diagnosis**			
< 2cm	122 (58.7%)	80 (57.1%)	42 (61.8%)
≥ 2 cm	86 (41.3%)	60 (42.9%)	26 (38.2%)
**Nodal status at time of diagnosis**			
Node-negative	163 (78.4%)	113 (80.7%)	50 (73.5%)
Node-positive	45 (21.6%)	27 (19.3%)	18 (26.5%)
**Tumour differentiation**			
G1	61 (29.3%)	42 (30.0%)	19 (27.9%)
G2	128 (61.5%	84 (60.0%)	44 (64.7%)
G3	19 (9.1%)	14 (10.0%)	5 (7.4%)
**ER status**			
ER positive (≥ 1%)	205 (98.6%)	137 (97.9%)	68 (100.0%)
ER negative (< 1%)	3 (1.4%)	3 (2.1%)	- -
**PgR status**			
PgR positive (≥ 1%)	177 (85.1%)	116 (82.9%)	61 (89.7%)
PgR negative (< 1%)	31 (14.9%)	24 (17.1%)	7 (10.3%)
**HR status (ER, PgR combined**)			
HR positive	208 (100.0%)	140 (100.0%)	68 (100.0%)
HR negative	- -	- -	- -
**HER2 Status**			
HER2 negative	208 (100.0%)	140 (100.0%)	68 (100.0%)
HER2 positive	- -	- -	- -
**IHC types**			
HR positive and HER2 negative	208 (100.0%)	140 (100.0%)	68 (100.0%)
HER2 positive and HR positive	- -	- -	- -
HER2 positive and HR negative	- -	- -	- -
TNBC	- -	- -	- -

Supplementary Table S 3 Univariate and multivariate analysis of RFI (A) and OS (B) considering PIK3CA mutation status

A

Group		sample size (n)	events (n)	RFI [%]	95% CI	Hazard ratio univariate RFI	95% CI	p-value	Hazard ratio multivariate RFI	95% CI	p-value
**ALL***		1,123	103								
	wiltype	823	79	89.8	87.6-92.0						
	mutated	300	24	90.9	87.4-94.4	0.867	0.554-1.359	0.535	1.193	0.752-1.894	0.454
**HR positive, any HER2 ****		935	58								
	wiltype	656	37	93.8	91.6-96.0						
	mutated	279	21	91.5	88.0-95.0	1.468	0.866-2.489	0.154	1.640	0.958-2.807	0.071
**HR positive, HER2 negative ****		824	49								
	wiltype	565	31	94.0	91.8-96.2						
	mutated	259	18	92.1	88.6-95.6	1.410	0.797-2.497	0.238	1.532	0.861-2.727	0.147
**HR positive, HER2 negative, AIs treated only****		208									
	wiltype	140	5	96.0	92.7-99.3						
	mutated	68	8	85.7	76.5-95.0	**4.231**	1.409 – 2.699	**0.010**	**4.391**	1.385-13.920	**0.012**
**HR positive, HER2 negative, TAM treated only****		192									
	wiltype	137	9	92.8	92.0-93.6						
	mutated	55	4	91.9	84.3-99.5	1.159	0.357-3.764	0.806	1.17	0.357-3.836	0.796
**HR negative, any HER2 ****		188	46								
	wiltype	167	43	72.9	66.0-79.8						
	mutated	21	3	84.4	68.1-99.9	0.514	0.159-1.660	0.266	0.493	0.152-1.597	0.238
**TNBC ****		128	33								
	wiltype	113	31	71.5	63.1-79.9						
	mutated	15	2	84.0	63.2-99.9	0.446	0.107-1.866	0.269	0.434	0.103-1.822	0.254
**HER2 positive, any HR ****		171	22								
	wiltype	145	18	86.6	80.7-92.5						
	mutated	26	4	84.6	70.7-98.5	1.144	0.387-3.380	0.808	1.519	0.505-4.568	0.457

B

Group		sample size (n)	events (n)	OS [%]	95% CI	Hazard ratio univariate OS	95% CI	p-value	Hazard ratio multivariate OS	95% CI	p-value
**ALL ***		1,123	129								
	wiltype	823	98	87.2	84.8-89.6						
	mutated	300	31	88.2	84.3-92.1	0.870	0.581-1.303	0.500	1.081	0.714-1.638	0.712
**HR positive, any HER2 ****		935	86								
	wiltype	656	57	90.5	88.1-92.9						
	mutated	279	29	88.1	84.0-92.2	1.221	0.782-1.906	0.380	1.366	0.867-2.152	0.179
**HR positive, HER2 negative ****		824	70								
	wiltype	565	44	91.5	89.1-93.9						
	mutated	259	26	88.6	84.5-92.7	1.322	0.816-2.142	0.257	1.365	0.837-2.227	0.212
**HR positive, HER2 negative, AIs treated ****		208									
	wiltype	140	15	85.2	79.6-91.4						
	mutated	68	15	73.5	63.0-85.8	**2.373**	1.172-4.80	**0.016**	**2.120**	1.021-4.404	**0.044**
**HR positive, HER2 negative, TAM treated only****		192									
	wiltype	137	7	94.5	90.6-98.4						
	mutated	55	4	91.7	83.9-99-5	1.524	0.446-5.208	0.501	1.601	0.463-5.533	0.457
**HR negative, any HER2 ****		188	56								
	wiltype	167	49	74.3	67.4-81.2						
	mutated	21	7	89.5	75.8-99.9	0.354	0.086-1.462	0.151	0.323	0.078-1.340	0.120
**TNBC ****		128	30								
	wiltype	113	28	74.3	66.1-82.5						
	mutated	15	2	85.7	77.8-99.9	0.483	0.115-2.027	0.320	0.451	0.107-1.903	0.278
**HER2 positive, any HR ****		171	29								
	wiltype	145	26	80.9	74.2-87.6						
	mutated	26	3	86.5	72.2-99.9	0.604	0.183-10.996	0.409	0.743	0.223-2.471	0.627

adjusted to:
* Tumour size, nodal status, grading, HR-status, HER2 Status, PIK3CA mutation status
** Tumour size, nodal status, grading, PIK3CA mutation status
bold: significant
